# Risk factors and outcome analysis after surgical management of ventricular septal rupture complicating acute myocardial infarction: a retrospective analysis

**DOI:** 10.1186/s13019-015-0265-2

**Published:** 2015-05-04

**Authors:** Shih-Ming Huang, Shu-Chien Huang, Chih-Hsien Wang, I-Hui Wu, Nai-Hsin Chi, Hsi-Yu Yu, Ron-Bin Hsu, Chung-I Chang, Shoei-Shen Wang, Yih-Sharng Chen

**Affiliations:** 1 Departments of Surgery, Buddhist Tzu Chi General Hospital, Dalin Branch, Chiayi, Taiwan; 2 Departments of Surgery, National Taiwan University Hospital, National Taiwan University College of Medicine, Taipei, Taiwan

**Keywords:** Ventricular septal rupture, Acute myocardial infarction, EuroSCORE, Left ventricular function

## Abstract

**Background:**

Ventricular septal rupture (VSR) is an uncommon but well-recognized mechanical complication of acute myocardial infarction (AMI). The outcome of VSR remains poor even in the era of reperfusion therapy. We reviewed our experience with surgical repair of post-infarction VSR and analyzed outcomes in an attempt to identify prognostic factors.

**Methods:**

From October 1995 to December 2013, data from 47 consecutive patients (mean age, 68 ± 9.5 years) with post-infarction VSR who underwent surgical repair at our institute were retrospectively reviewed. The preoperative conditions, morbidity and surgical mortality were analyzed. Multivariate analysis was subsequently carried out by constructing a logistic regression model in order to identify independent predictors of postoperative mortality. Long term survival function were estimated using the Kaplan-Meier method and compared using the log-rank test.

**Results:**

Percutaneous coronary intervention was performed in 17 (36.2%) patients, intra-aortic balloon pump (IABP) was used in 34 (72.3%), and six (12.8%) were supported with extracorporeal membrane oxygenation (ECMO) preoperatively. Forty-one (87.2%) patients received emergent surgical treatment. Concomitant coronary artery bypass grafting was performed in 27 (57.4%) patients.

Operative mortality was 36.2% (17 of 47). The survival rate was 59.3% with concomitant CABG and 70% without concomitant CABG (p = 14). Multivariate analysis revealed that the survivors had higher preoperative left ventricular ejection fractions (LVEFs) compared with those who died (51 ± 13.7% vs. 36.6 ± 6.4% , respectively; p < 0.001) and lower European system for cardiac operative risk evaluation II (EuroSCORE II) (22.9 ± 14.9 vs. 38.3 ± 13.9, respectively; p < 0.001). The patients receiving total revascularization has long term survival benefit (p = 0.028).

**Conclusions:**

Post-infarction VSR remains a serious and challenging complication of AMI in the modern surgical era. The EuroSCORE II can be used for an approximate prediction of operative mortality. Preserved LVEF was associated with better prognosis, while the need for postoperative RRT was associated with higher early and late mortality. Besides, the strategy of total revascularization should be applied to ensure long-term survival benefit.

## Background

Ventricular septal rupture (VSR) is a fatal complication following acute myocardial infarction (AMI). The incidence of VSR complicating AMI was 1-3% in the era prior to widespread reperfusion therapy [1]. Since the introduction of thrombolytic therapy, the incidence has declined to approximately 0.3% [2]. After undergoing a primary percutaneous coronary intervention (PCI), VSR was reported to occur in 0.23-0.71% of patients [3-5].

Treatment of VSR after AMI is a surgical challenge and the surgical mortality rate of post-infarction VSR remains high. A reported mortality of 34%-54% has remained relatively constant during the past two decades [6-9]. We reviewed our experience of post-infarction VSR and analyzed the surgical outcomes in an attempt to identify risk factors associated with mortality.

## Methods

This study was approved by the Institutional Review Board of our institute (201405033RINA) and informed consent was waived. During the period from 1995 to 2013, patients with a diagnosis of AMI complicated by VSR were retrieved from the registration database of our hospital using ICD-9 codes (ICD-9 code 429.71, I232). The demographic data, clinical presentation, surgical management, and outcomes were reviewed. Patients who underwent concomitant coronary revascularization procedures were also included.

### Surgical technique

**Figure 1:**
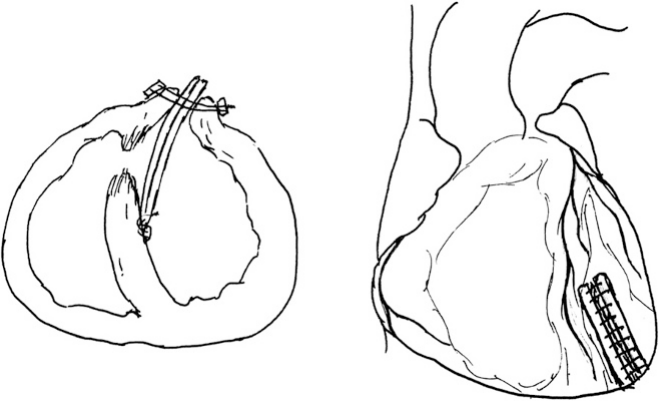
VSR repair is performed with the concept of “infarct exclusion”. The LV incision is made parallel to the left anterior descending artery, the patch is sutured on the healthy myocardium using 3–0 pledgetted prolene interrupted suture, and the LV ventriculotomy is closed, excluding the infarcted myocardium from the high pressure of the LV cavity.

The operation was performed with standard cardiopulmonary bypass (CPB). The VSR repair was performed with the concept of “infarct exclusion” [10,11], as shown in Figure 1.

The left ventricle was incised to expose the location of the VSR, and margins of the infarcted muscle were identified. If it was difficult to expose the location of VSR, such as posterior ventricular septal rupture, the longitudinal right atriotomy was performed to expose the location and margin. The patch (either xenograft or prosthetic) was tailored in the shape of the left ventricular infarction and then sutured to a portion of the non-infarcted interventricular septum with intermittent pledgetted 3–0 polypropylene suture. After the VSR was repaired, the LV was closed with 2–0 prolene with felt strips for enforcement. According to our strategy, the application of complete revascularization was not carried out in all patients. If PCI was not performed before the operation or culprit-only percutaneous coronary intervention was performed, concomitant coronary artery bypass grafting (CABG) to the stenotic vessels was undertaken, if feasible.

### Variables

The demographic characteristics, medical co-morbidities (smoking history, hypertension, diabetes mellitus, previous cerebrovascular accident, and renal function), medical acuity (prodromal angina, left ventricular ejection fraction (LVEF), and Killip classification), the location of infarction, site of septal rupture, and preoperative peak cardiac enzymes were obtained by chart review. Prodromal angina was defined as typical chest pain episodes (either at rest or upon effort) persisting < 30 minutes and occurring within 24 hours before the onset of the AMI [12].

The use of early PCI (≤6 hours after AMI), intra-aortic balloon pump (IABP), concomitant surgical procedures, and calendar year of operation were also included as variables for analysis.

The surgical condition was defined according to the clinical status of the patient at the time of surgery [6]. Elective status was defined as routine admission for the operation. Emergent status was defined as surgery for those patients with hemodynamic instability, including hypotension or tachyarrhythmia, even under use of inotropic agents or placement of an intra-aortic balloon pump.

The ratio of pulmonary to systemic blood flow was calculated using the Swan-Ganz catheter data, i.e., Qp/Qs = S_Ao_O_2_ – S_RA_O_2_/S_PV_O_2_ – S_PA_O_2_, where Ao = aorta, RA = right atrial, PV = pulmonary vein, and PA = pulmonary artery. SaO_2_ was substituted for S_PV_O_2_ if SaO_2_ was not available.

To determine if the European system for cardiac operative risk evaluation (EuroSCORE) could predict operative mortality in patients with myocardial infarction complicating VSR, we calculated the EuroSCORE II for each patient [13].

### Early outcomes

The early outcome was operative mortality, defined as death from any cause in-hospital or within 30 days of the index operation [6]. Outcome data for operative mortality and subsequent survival were obtained for all patients from hospital medical records until March 2014.

The secondary outcome included length of ICU stay and morbidities such as postoperative pneumonia, acute kidney injury (AKI) after operation requiring renal replacement therapy (RRT), cerebrovascular accident, re-exploration for bleeding, and residual VSR.

AKI was defined using the KDIGO (Kidney Disease/Improving Global Outcomes) clinical practice guideline. Stage 1, according to this guideline was defined as an increase in serum creatinine of ≥ 0.3 mg/dL (≥26.5 μmol/L) within 48 hours or an increase in serum creatinine of ≥ 1.5 times baseline (which was known or presumed to have occurred within the prior seven days), or urine output < 0.5 mL/kg/h for 6 hours [14]. Patients that met the definition for AKI were staged according to severity, including an increase in serum creatinine or reduction in urine output, as shown in Table 1.

**Table 1:** **Staging of acute kidney injury based on KDIGO Clinical Practice Guidelines for Acute Kidney Injury [** 14 **]**

Stage	Serum creatinine	Urine output
1	1.5–1.9 times baseline	<0.5 ml/kg/h for 6–12 hours
	OR	
	≥0.3 mg/dl (≥26.5 mmol/l) increase	
2	2.0–2.9 times baseline	<0.5 ml/kg/h for ≥12 hours
3	3.0 times baseline	<0.3 ml/kg/h for ≥24 hours
	OR	OR
	Increase in serum creatinine to ≥4.0 mg/dl (≥353.6 mmol/l)	Anuria for ≥12 hours
	OR	
	Initiation of renal replacement therapy	
	OR,	
	In patients <18 years, decrease in eGFR to <35 ml/min per 1.73m^2^	

Adapted from [14]: Improving Global Outcomes (KDIGO) Acute Kidney Injury Work Group. KDIGO Clinical Practice Guideline for Acute Kidney Injury. Section 2: AKI definition. Table 2. Staging of AKI. Kidney International Supplements 2012;2:19. http://www.kdigo.org/clinical_practice_guidelines/pdf/KDIGO%20AKI%20Guideline.pdf.

It remains unclear whether patients with AKI after cardiac surgery would benefit from the early institution of pharmacologic agents or the early initiation of RRT [15]. In current practice, the decision to start RRT is based on clinical features of volume overload and biochemical features of solute imbalance (azotemia, hyperkalemia, or severe acidosis). Postoperative RRT was required in approximately 1-8% of these patients after cardiac surgery and this treatment was associated with increased mortality and hospitalization costs [16,17].

### Late outcome

Follow-up data were obtained by consulting the hospital medical records and by telephoning patients or their families.

### Statistical analysis

Summary statistics for outcomes and baseline patient characteristics were expressed as percentages for categorical variables and as mean ± standard deviation (SD) for continuous variables. The Chi-square test was used to compare categorical variables, whereas the Student t-test was used for continuous variables. Subsequently, multivariate analysis was carried out by constructing a logistic regression model with the above-mentioned variables in order to identify independent predictors of postoperative mortality.

Survival functions were estimated using the Kaplan-Meier method and compared using the log-rank test. We calculated the risk ratios at specific points, using the estimated rates of survival for patients with/without complete revascularization. A multivariate Cox proportional hazard analysis was performed to identify the independent factors affecting long-term survival. A two-tailed *p-*value less than 0.05 was used to indicate statistical significance.

## Results

### Incidence

During the period from 1995 to 2013, there were 4761 patients diagnosed with AMI and admitted to our hospital. Among them, 47 (0.98%) patients had AMI complicated by VSR.

### Clinical characteristics

The patients’ demographic characteristics are presented in Table 2. The average age at operation was 68.9 ± 9.5 years and men accounted for 59.6% of the patients. Most of the patients (85.1%) had one or more comorbidities of which hypertension was the most common (61.7%), followed by diabetes mellitus (44.7%).

**Table 2:** **Characteristics of the 47 patients with ventricular septal rupture after acute myocardial infarction**

Profile		Mean ± SD or No.(%)
**Demographics**		
	Age, years	68.9 ± 9.5
	Male sex	28(59.6%)
	Body weight	62.5 ± 10.2
**Comorbidities**		
	Hypertension	29(61.7%)
	Diabetes mellitus	21(44.7%)
	Smoking	14(29.8%)
	Dialysis	4(8.5%)
	Estimated Creatinine Clearance Rate [=(140-age)xBWx0.85 if woman/72xScr]	44.2 ± 20.6
	History of Cerebrovascular accident	7(14.9%)
**Acuity**		
	Pre-OP mechanical support	
	IABP	34(72.3%)
	ECMO	6(12.8%)
	Prodromal Angina	29(61.7%)
	Killip class	
	I	6(12.8%)
	II	7(14.9%)
	III	15(31.9%)
	IV	19(40.4%)
	Coronary artery disease	
	1-V-D	16(34%)
	2-V-D	10(21.3%)
	3-V-D	21(44.7%)
	Left ventricular Ejection fraction(%)	45.8 ± 13.5
	Cardiac emzyme	
	CK	1392.1 ± 1208.3
	CK-MB	115.9 ± 127.0
	EuroSCORE II	28.5 ± 16.2
**VSR characteristics**		
	Anterior MI	42(89.4%)
	Apical rupture	36(76.6%)
	Ventricular Aneurysm	20(42.6%)
	Qp/Qs	3.0 ± 1.6
**Revascularization**		
	PCI	17(36.2%)
	Thrombolysis	12(25.5%)
**OP characteristics**		
	Repair from MI, days	6.3 ± 10.4
	Emergency surgery	41(87.2%)
	Concomitant CABG	27(57.4%)
	CPB time	193.9 ± 50.4
	AXC time	113 ± 46.9

*Abbreviations:IABP* intra-aortic balloon pump; *ECMO* extracorporeal membrane oxygenation; *V-D* vessel disease; *CK* creatinine kinase; *CK-MB* creatinine kinase MB fraction; *EuroSCORE II* European system for cardiac operative risk evaluation II; *Qp/Qs* ratio of pulmonary to systemic blood flow; *PCI* percutaneous coronary intervention; *MI* myocardial infarction; *CABG* coronary artery bypass grafting; *CPB* cardiopulmonary bypass; *AXC* aortic cross-clamp.

**Figure 2:**
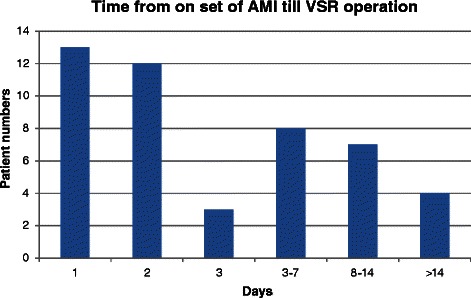
Based on the time between diagnosis of myocardial infarction and surgery, most surgeries are performed as urgent or emergent operations after diagnosis is confirmed.

The time between diagnosis of AMI and VSR repair was 5.3 ± 10.4 days (Figure 2). Emergency surgery was performed in 41(87.2%) patients. Before operation, 34 patients (72.3%) were supported by IABP and the majority of patients at the time of diagnosis of AMI were Killip class III (n = 15, 31.9%) or class IV (n = 19, 40.4%). Anterior myocardial infarction (n = 42, 89.4%) was the most common location of myocardial infarction. The apical VSR (n = 36, 76.6%) was the most common VSR location, while there were 11 patients with the posterior VSR. The average LVEF before operation was 45.8 ± 13.5% (Table 2).

Before operation, revascularization procedure was performed in 18 patients. Among them, 17 patients received PCI and 12 received thrombolysis.

During the operation, all had VSR repaired with patches and pledgetted-prolene suture; no closure devices were used. Concomitant CABG was performed in 27 patients (57.4%). The duration of CPB was 194 ± 50 minutes and aortic clamp-time was 113 ± 47 minutes, as shown in Table 2.

### Early outcomes

The operative mortality was 36.2% (n = 17) and the median ICU stay of the survivors was 15 days.

Postoperative morbidity was common, including cerebral vascular accident in five (10.6%) patients, AKI requiring RRT in 21 (48.8%), pneumonia in 20 (42.6%), and heart block in seven (14.9%). Eleven (23.4%) patients required re-exploration for mediastinal bleeding. Residual ventricular septal defect was noted in 16 patients (34%), including eight who were diagnosed during ICU admission (Table 3).

**Table 3:** **Post-operative outcome of the 40 patients with ventricular septal rupture**

Postoperative outcomes	Mean ± SD or No.(%)
**Length of hospitalization, days**	44.9 ± 36.0
**ICU stay of survivors, days**	24.9 ± 27.3
**Cerebrovascular accident**	5(10.6%)
**Renal replacement therapy**	21(48.8%)
**Pneumonia**	20(42.6%)
**Heart block**	7(14.9%)
**Reexploration for bleeding**	11(23.4%)
**Residual VSD**	16(34%)

*Abbreviations: ICU* intensive care unit; *VSD* ventricular septal defect; *SD* standard deviation.

From Table 4, age, gender, preoperative co-morbidity, Killip class, number of diseased coronary arteries, location of VSR, Qp/Qs, peak cardiac enzymes, and revascularization before operation were not significantly different between survivors and non-survivors. The operative mortality rate did not vary by the interval between diagnosis of AMI and repair (p = 0.19). There was no significant difference in the duration of aortic cross-clamping between survivors and non-survivors (109 ± 44 minutes vs. 123 ± 55 minutes, respectively, p = 0.424). In addition, the use of prior coronary reperfusion therapy and concomitant CABG were not significantly different between survivors and non-survivors (76.7% vs. 74.5%, respectively; p = 0.646).

**Table 4:** **Comparison of clinical characteristics between Survivors and Nonsurvivors**

Variable	Survivors (N = 30)	Nonsurvivors (N = 17)	p value	Multi-varible p
**Demographics**				
Age, years	68.0 ± 7.9	70.5 ± 11.9	0.402	
Male sex	18(60%)	10(58.8%)	0.937	
Body weight	62 ± 10.4	63.4 ± 10.0	0.661	
**Comorbidities**				
Hypertension	21(70%)	8(47.1%)	0.120	
Diabetes mellitus	15(50%)	6(35.3%)	0.330	
Smoking	7(23.3%)	7(41.2%)	0.199	
Dialysis	2(6.7%)	2(11.8%)	0.547	
Estimated Creatinine Clearance Rate	45.9 ± 18.2	41.3 ± 24.6	0.467	
History of Cerebrovascular accident	4(13.3%)	3(17.6%)	0.690	
**Acuity**				
Pre-operative mechical support				
IABP	20(66.7%)	14(82.4)	0.248	
ECMO	4(13.3%)	2(11.8%)	0.877	
Prodromal Angina	21(70%)	8(47.1%)	0.120	
Killip class				
I	4(13.3%)	2(11.8%)	0.877	
II	5(16.7%)	2(11.8%)	0.650	
III	9(30%)	6(35.3%)	0.708	
IV	12(40%)	7(41.2%)	0.937	
Coronary artery disease				
1-V-D	12(40%)	4(23.5%)	0.252	
2-V-D	4(13.3%)	6(35.3%)	0.077	
3-V-D	14(46.7%)	7(41.2%)	0.716	
LV Ejection fraction	51 ± 13.7	36.6 ± 6.4	0.001	0.00.3
Cardiac emzyme				
CK	1436.4 ± 1287.2	1324.3 ± 1116.5	0.784	
CK-MB	129.3 ± 142.6	95.2 ± 99.5	0.425	
EuroSCORE II	22.9 ± 14.9	38.3 ± 13.9	0.001	0.183
**Revascularization**				
PCI	11(36.7%)	6(35.3%)	0.925	
Thrombolysis	7(23.3%)	5(29.4%)	0.646	
**AMI/VSD characteristics**				
Anterior MI	27(90%)	15(88.2%)	0.850	
Apical rupture	24(80%)	12(70.6%)	0.464	
Ventricular Aneurysm	15(50%)	5(29.4%)	0.17	
Qp/Qs	2.8 ± 1.6	3.2 ± 1.68	0.538	
**Operative characteristics**				
Repair from MI, days	7.8 ± 12.5	3.7 ± 3.7	0.190	
Emergency surgery	24(80%)	17(100%)	0.048	0.093
Concomitant CABG	16(53.3%)	11(64.7%)	0.449	
CPB time	189.1 ± 44.2	205.7 ± 64.2	0.364	
AXC time	109.1 ± 44.0	122.7 ± 54.6	0.424	
**Cohort effect (2007-2013)**	15(50%)	3(17.6%)	0.028	0.649
**Post-opearative outcome**				
Cerebrovascular accident	2(6.7%)	3(17.6%)	0.241	
Renal replacement therapy	8(28.6%)	13(86.7%)	0.001	0.001
Pneumonia	11(36.7%)	9(52.9%)	0.278	
Heart block	5(16.7%)	2(11.8%)	0.650	
Reexploration for bleeding	4(13.3%)	7(41.2%)	0.030	0.322
Residual VSD	9(30%)	7(41.2%)	0.437	

*Abbreviations: IABP* intra-aortic balloon pump; *ECMO* extracorporeal membrane oxygenation; *V-D* vessel disease; *CK* creatinine kinase; *CK-MB* creatinine kinase MB fraction; *EuroSCORE II* European system for cardiac operative risk evaluation II; *Qp/Qs* ratio of pulmonary to systemic blood flow; *PCI* percutaneous coronary intervention; *MI* myocardial infarction; *CABG* coronary artery bypass grafting; *CPB* cardiopulmonary bypass; *AXC* aortic cross-clamp; *VSD* ventricular septal defect.

The survivors had a higher LVEF than the non-survivors (51 ± 13.7% vs. 36.6 ± 6.4%, p = 0.001). The patients who received emergent surgical repair (n = 41) had a higher mortality than those who received elective repair (n = 6) (41.5% vs. 0%, respectively; p = 0.048).

**Figure 3:**
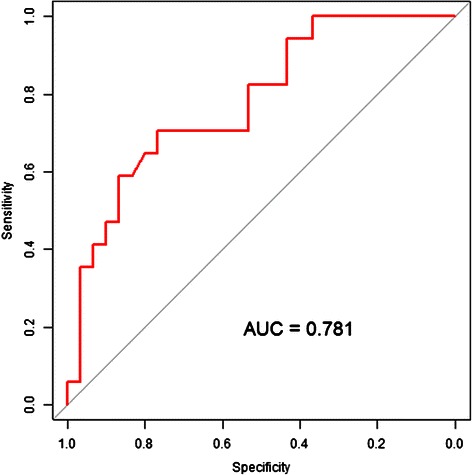
The receiver operating characteristic (ROC) curve shows the relationship between EuroSCORE II and operative mortality. By ROC curve analysis, EuroSCORE II has excellent discriminatory power for operative mortality, with an area under the curve (AUC) of 0.781.

The EuroSCORE II was significantly higher in the non-survivors than the survivors (38.3 ± 13.9 vs. 22.9 ± 14.9, respectively, p =0.001). A higher EuroSCORE II predicted a higher possibility of operative mortality. In this study population, EuroSCORE II had excellent discriminatory power for operative mortality, with an area under the curve (AUC) of 0.781(Figure 3).

Postoperative morbidity was common in both survivors and non-survivors, while the non-survivors required more frequent postoperative dialysis (86.7% vs. 28.6%, respectively; p = 0.001; Table 4). The operative mortality over the most recent 8 years (2006–2013) was 16.67% (3/18), much lower than previous 11 years(1995–2005) operative mortality [48.28% (14/29); p = 0.028].

Significant predictors of operative mortality based on univariate analysis included LVEF, EuroSCORE II, emergent surgical status, two consecutive period cohort effect, and postoperative RRT. Logistic regression for multivariate analysis showed that the LVEF and postoperative RRT were independent risk factors for operative mortality, as shown in Table 4.

### Late outcome

**Figure 4:**
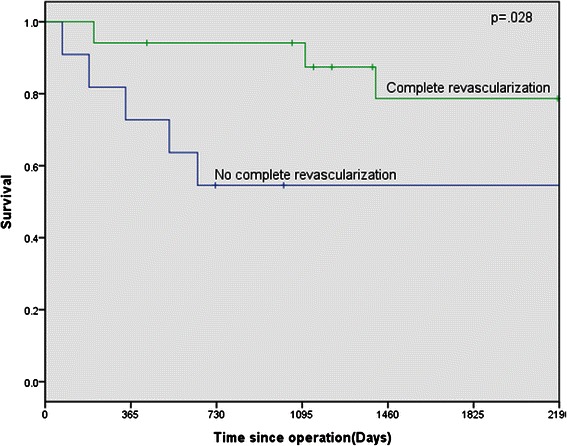
Kaplan-Meier survival curve. Log-rank p = 0.028.

Follow-up was complete in 93.3% of patients with two patients lost to follow-up. The overall average follow-up period was 99.1 months (range, 72.8-125.3) for the 28 patient survivors. Figure 4 shows the Kaplan-Meier estimate of overall survival, including operative deaths. Overall survival at 6 years was 41.1 ± 2.2%.

Based on multivariate Cox regression analysis, the factor that had a significant positive impact on late survival was total revascularization of stenotic coronary arteries. Figure 4 demonstrates an increasing survival benefit for the patients receiving total revascularization (p = 0.028).

## Discussion

This study examined outcomes in 47 patients with VSR following AMI who underwent surgical repair over a 19 year period at our institution. The observed incidence of VSR complicating AMI was 0.98%, which was lower than the incidence quoted from pre-thrombolytic era studies, but slightly higher than the incidence (0.2 ~ 0.4%) reported in the Global Utilization of Streptokinase and t-PA for Occluded Coronary Arteries (GUSTO-I) trial of more than 41,000 patients treated for myocardial infarction in the thrombolytic era [2]. However, since our study was performed at a tertiary medical center, our patients may have had a higher disease severity. In addition, some patients were referred after diagnosis of VSR, which could have increased our observed incidence.

Our operative mortality rate was 36.2% which is consistent with the 34-54% operative mortality rate seen in previous studies [6-9]. In addition, we found that the operative mortality of VSR complicating AMI was much lower during the previous 8 years (2006–2013) (3/18, 16.67%) compared with the previous 11 years (1995–2005) 14/29, 48.28%, respectively; p = 0.028). In the study by Figueras et al., in-hospital deaths for patients with AMI and cardiac rupture declined over time and most were due to cardiac causes [5].

For our whole cohort, the predicted mortality rate by the EuroSCORE II was 28.5 ± 16.2%. We observed a higher mortality than predicted by the EuroSCORE II. The non-survivors had a higher EuroSCORE II than the survivors (38.4 ± 13.9% vs. 22.9 ± 14.9, respectively; p = 0.001).

The EuroSCORE II scoring system is commonly applied in cardiac surgery, including CABG and valvular surgery. EuroSCORE II reflects better current surgical performance and offers a new quality standard to evaluate clinic outcomes [13,18]. The discriminatory ability of the EuroSCORE II for operative mortality by area under the curve (AUC) was 0.781 in our study. Although EuroSCORE II’s predicted operative mortality was overestimated in previous studies [19,20], the EuroSCORE II did not overestimate mortality in this study and can reflect the mortality risk in a small group of patients.

In a previous study [21], higher postoperative mortality, longer intensive care unit stays, and longer hospital stays were observed in patients who underwent CABG with lower LVEF. Similar to the findings of Philip et al. [22], our data showed that preserved LVEF had a positive impact on early survival. In post-infarct VSR, cardiogenic shock is due, in part, to a decreased myocardial contractility in the infarcted area, and is also secondary to the presence of a left-to-right shunt. Thus, the low LVEF might imply less myocardial reserve after VSR repair and, thus, may have contributed towards a higher mortality rate.

The patents in our cohort who underwent a lower-risk elective procedure at a later time achieved better survival although the difference was not significant upon multivariate analysis. Medical therapy or mechanical support (such as IABP) can stabilize the hemodynamic status of patients and with careful monitoring allow elective surgery at a later time. A longer interval before surgery has been associated with improved survival [1,23,24]. Delayed elective repair allows for myocardial scar tissue formation which may facilitate the technical aspects of VSR repair. Despite this, a longer interval between diagnosis of AMI and repair did not significantly improve survival.

We also observed that postoperative AKI requiring dialysis was independently associated with mortality and these same findings have been previously reported [22]. Renal dysfunction in the surgical patient is usually multifactorial. The most common cause is ATN as a result of hypoxic damage to nephrons in the medullary region of the kidney secondary to hypotension, hypovolemia, and dehydration. Patients with post-infarction VSR experienced cardiogenic shock, CPB, and/or circulatory arrest. Postoperative AKI is a serious condition that carries a considerable mortality. The incidence of acute renal failure after CABG requiring dialysis is less than 2%, but in such cases, the mortality varies between 23 and 88% [25-27]. Therefore, prevention of organ injury (including prevention of shock and preservation of organ perfusion) is important in improving survival.

Lundblad et al. [28] found that concomitant CABG during VSR repair reduces both early and late mortality when compared with patients with unbypassed coronary artery disease. In a review of recent literature, Perotta et al. [29] also reported an improvement in mortality rates in those patients who had undergone CABG. These results were applied to patients with multi-vessel disease where complete myocardial revascularization was achieved by bypassing all stenotic coronary arteries supplying noninfarcted areas. Our study found that concomitant CABG did not confer a protective effect in early mortality, but a pronounced survival benefit was provided by the total revascularization during long-term follow-up.

### Limitations

Our study had several limitations. Due to its retrospective design, we were unable to certify that all potential confounding factors had been record. Due to the relatively rare occurrence of post-infarction VSR, the small sample size underpowered the statistical analysis and could have limited the number of statistically significant variables. During the study period, the revascularization by PCI and surgical techniques may have improved as our experience accumulated. Our single center experience may not be applicable to other institutes.

## Conclusions

Post-infarction VSR carries significant mortality (36.2%) despite aggressive surgical management. The surgical results have shown improvement in recent years (2006–2013). The EuroSCORE II can be used to make an approximate prediction of operative mortality. Preserved LVEF had a beneficial effect on early prognosis, while the need for postoperative RRT was associated with higher early and late mortality. The strategy of total revascularization should be applied to ensure long-term survival benefit.
